# Selection and Genetic Analysis of High Polysaccharide-Producing Mutants in *Inonotus obliquus*

**DOI:** 10.3390/microorganisms12071335

**Published:** 2024-06-29

**Authors:** Lanlan Hua, Hongling Shi, Qing Lin, Haozhong Wang, Yan Gao, Jun Zeng, Kai Lou, Xiangdong Huo

**Affiliations:** 1 Institute of Microbiology, Xinjiang Academy of Agricultural Sciences, Urumqi 830091, China; 2 Xinjiang Laboratory of SpecialEnvironmental Microbiology, Urumqi 830091, China; 15276793610@163.com (L.H.); 15509947314@163.com (H.S.); qinglinxj@163.com (Q.L.); gaoyan19790826@outlook.com (Y.G.); leo924.student@sina.com (J.Z.); 3 College of Life Science and Technology, Xinjiang University, Urumqi 830046, China

**Keywords:** ARTP, genome resequencing, polysaccharide

## Abstract

*Inonotus obliquus*, a medicinal fungus, has garnered significant attention in scientific research and medical applications. In this study, protoplasts of the *I. obliquus* HS819 strain were prepared using an enzymatic method and achieved a regeneration rate of 5.83%. To enhance polysaccharide production of *I. obliquus* HS819, atmospheric and room temperature plasma (ARTP) technology was employed for mutagenesis of the protoplasts. Through liquid fermentation, 32 mutant strains exhibiting diverse characteristics in morphology, color of the fermentation broth, mycelial pellet size, and biomass were screened. Secondary screening identified mutant strain A27, which showed a significant increase in polysaccharide production up to 1.67 g/L and a mycelial dry weight of 17.6 g/L, representing 137.67% and 15% increases compared to the HS819 strain, respectively. Furthermore, the fermentation period was reduced by 2 days, and subsequent subculture cultivation demonstrated stable polysaccharide production and mycelial dry weight. The genome resequencing analysis of the HS819 strain and mutant strain A27 revealed 3790 InDel sites and mutations affecting 612 functional genes associated with polysaccharide synthesis. We predict that our findings will be helpful for high polysaccharide production through genetic engineering of *I. obliquus*.

## 1. Introduction

*Inonotus obliquus*, a rare medicinal fungus, has become a valuable resource in the pharmaceutical and functional food industries. The fungus contains various bioactive substances, including triterpenoids, polysaccharides, steroids, polyphenols, and melanin [1]. Polysaccharides show a wide range of therapeutic potential, including anticancer [2], hypoglycemic [3], anti-inflammatory [4], antioxidant [5], anti-fatigue [6], and antiviral [7] activities. Although extensive research has been conducted on *I. obliquus* polysaccharides, current studies on improving polysaccharide production and biomass have mainly focused on optimizing the composition of culture media and fermentation conditions, including carbon source optimization [8], the addition of inducers [9], and controlling the aeration rate [10]. However, these methods have limited effectiveness and high costs. Modern genetic engineering technologies have been widely applied in microbial breeding, but these are severely limited by the unknown genetic backgrounds of the experimental strains. Random mutagenesis techniques have shown significant advantages in microbial improvement and breeding. They do not rely on host cell genetic information and can generate mutant libraries with genetic diversity. This is relatively simple and cost-effective [11]. Random mutagenesis methods include chemical mutagenesis, such as diethyl sulfate (DES) [12] and N-methyl-N’-nitro-N-nitrosoguanidine (NTG) [13], as well as physical mutagenesis, such as ultraviolet (UV) radiation [14] and Co60-γ rays [15]. However, these conventional methods may cause potential permanent harm to the environment and to users [16]. Atmospheric and room temperature plasma (ARTP) technology, as a novel physical mutagenesis method, has been successfully applied to generate various fungal mutants, owing to its advantages of easy operation, high safety, high mutagenic throughput, high total and positive mutation rates, and high genetic stability of mutants [17], such as *Phellinus baumii* [18], *Grifola frondosa* [19], and *Chlorella pyrenoidosa* [20]. ARTP can penetrate cell walls and membranes, causing maximum DNA damage to individual cells while maintaining cell viability, thereby inducing mutations and altering metabolism [21,22]. It operates at room temperature (25–40 °C), thus avoiding potential heat damage to thermosensitive microorganisms. This study represents the first attempt to utilize ARTP-induced mutants to enhance the polysaccharide productivity of *I. obliquus*.

Fungal protoplasts, as single-cell materials for mutagenesis breeding, can improve breeding efficiency. For example, Zhang et al. [23] utilized ARTP technology to treat the protoplasts of *Pleurotus djamor*. Through liquid shake flask fermentation of the high laccase-producing positive mutants, mutant strain 51-4 with the highest Lac activity of 494.44 U/L was identified, representing an 86.36% increase compared to the wild-type strain, with superior growth and genetic stability. In another study, a superior strain M27, with a 25.3% increase in gibberellic acid production (2.38 g/L), was obtained by co-mutagenizing the protoplasts of *Fusarium fujikuroi* using ARTP and EMS (ethyl methanesulfonate) methods, maintaining high yields over 10 generations [24,25]. Currently, there are few reports on mutagenesis breeding of *I. obliquus*, mainly using mycelia as the mutagenic material [26]. However, direct mutagenesis of mycelium cells is not ideal, due to the protective effect of the cell wall, limited DNA damage, and effective self-repair for achieving desirable mutational effects and obtaining pure mutant strains, with no reported methods for spore production and collection under indoor conditions. Protoplasts, with only a cell membrane that is more susceptible to mutagenic agent damage, can partially replace spores, offering the potential to obtain high-producing polysaccharide-positive mutants.

Currently, the analysis of the structure and functional activities of polysaccharides from *I. obliquus* is continuously deepening. However, little research has been conducted on the key regulatory genes or enzymes involved in the biosynthesis of polysaccharides in *I. obliquus*. With the advent of the post-genomic era, omics technologies, including genomics, transcriptomics, and proteomics, have been widely applied to edible microorganisms to reveal the biosynthesis and regulation of intracellular polysaccharides, laying the foundation for the high production of active polysaccharides and the development of products [27]. For example, Wu et al. [28] discovered through RNA-seq analysis that MAPK, amino sugar and nucleotide sugar metabolism, peroxisomes, starch and sucrose metabolism, TCA cycle, glycolysis/gluconeogenesis KEGG pathway, glycosyltransferases, glycoside hydrolases and autophagy, and ubiquitin-mediated protein degradation play important roles in enhancing the production of extracellular polysaccharides in the *Ganoderma lucidum* Tween 80-treated group. Lin et al. [29] demonstrated through transcriptomic analysis that cpsA is a key gene involved in the biosynthesis of mannose and Cordyceps polysaccharides (CP), and its upregulation significantly enhances CP production. Yang et al. [30] validated, through HiSeq2500 transcriptomic analysis and qRT-PCR, that many genes involved in polysaccharide biosynthesis in *Poria cocos* encode key enzymes related to polysaccharide synthesis, including malZ, galA, SORD, gnl, and bglX. These findings suggest that omics analysis may be a feasible approach for uncovering the genes and key enzymes associated with intracellular polysaccharide production in *I. obliquus*.

This study represents the first utilization of the ARTP technique to induce protoplast mutation and screening for high polysaccharide-producing mutants in *I. obliquus*. Whole-genome resequencing and comparative analysis were performed on the high-producing mutants and the parental strain to elucidate the biosynthetic pathways and functional genes associated with polysaccharide overproduction in *I. obliquus*. This research lays the foundation for the rational regulation of polysaccharide synthesis and the cultivation of high-quality strains of *I. obliquus*.

## 2. Materials and Methods

### 2.1. Liquid Fermentation and Polysaccharide Determination

Stain HS819 of *I. obliquus* was provided by the Institute of Microbiology, Xinjiang Academy of Agricultural Sciences. Four 1 cm diameter mycelial plugs of *I. obliquus* cultured on CYM agar plates (2 g peptone, 4 g yeast extract, 20 g glucose, 0.5 g MgSO_4_, 0.48 g K_2_HPO_4_, 0.5 g KH_2_PO_4_, and 16 g agar) for 15 days were transferred into a 50 mL centrifuge tube. After grinding, 5 mL of sterile water was added and mixed. The mixture was then inoculated into 100 mL of CYM seed culture medium (2 g peptone, 4 g yeast extract, 20 g glucose, 0.5 g MgSO_4_, 0.48 g K_2_HPO_4_, and 0.5 g KH_2_PO_4_) and cultivated at 30 °C with shaking at 150 rpm for 4 days. Subsequently, the seed culture was transferred into 100 mL of fermentation medium (25.0 g glucose, 7.0 g bran, 10.0 g soybean meal, 8.0 g corn powder, 4 g yeast powder, 1 g KH_2_PO_4_, 0.5 g MgSO_4_, pH 6) with an inoculum size of 10% and cultivated at 30 °C with shaking at 150 rpm for 10 days.

The mycelia were filtered from the fermentation broth using gauze, washed with distilled water until clear, dried at 50 °C to a constant weight, and then weighed. The dried mycelia were crushed, distilled water (solid–liquid ratio: 1:40) was added, it was ultrasonicated at 50 °C for 25 min and centrifuged at 2248× *g* for 10 min, and the supernatant was retained. We repeated the above steps three times [31]. The supernatants were combined, and we added anhydrous ethanol to a final concentration of 75%. Then, we let it sit at 4 °C for 12 h, centrifuged at 4500 r/min for 10 min, discarded the supernatant, and retained the precipitate. We dissolved the precipitate in distilled water and measured the polysaccharide concentration [32] as follows: Polysaccharide content = Total sugar content-Reducing sugar content. The total sugar content was determined using the phenol-sulfuric acid method [33], and reducing sugar content was determined using the DNS method [34].

### 2.2. Preparation and Regeneration of Protoplasts

The *I. obliquus* cultured on CYM solid plates was transferred using a cork borer to obtain four 1 cm diameter fungal disks, which were then transferred to 50 mL centrifuge tubes. After grinding, 5 mL of sterile water was added and mixed thoroughly. Subsequently, the mixture was inoculated into 100 mL CYM seed culture medium and incubated at 30 °C with shaking at 150 rpm for 6 d to obtain mycelia for protoplast preparation. Under aseptic conditions, mycelia from liquid fermentation were filtered through filter paper and washed with distilled water before being transferred to EP tubes, then 600 µL of 0.6 mol/L mannitol was added, and the mycelia were homogenized using a hand-held homogenizer. The homogenate was centrifuged at 11,100× *g* for 10 min to remove the mannitol, leaving behind the precipitate. The collected mycelial homogenate was incubated with a pretreatment solution (0.5% mercaptoethanol dissolved in 1.0 mol/L phosphate buffer, pH 6.0) for 30 min, with the untreated homogenate serving as a control. The lytic enzyme purchased from the Guangdong Institute of Microbiology was separately added to the two groups of mycelial homogenates at a final enzyme concentration of 30 mg/mL. Enzymatic digestion was carried out at 30 °C for 1.5 h. After completion of enzymatic digestion, residual mycelial fragments were removed using a G3 sand core shot funnel. The remaining mycelial fragments were repeatedly washed with 0.6 mol/L mannitol and filtered through a G3 sand core funnel to collect the filtrate for obtaining a high concentration of protoplasts. The filtrate was then centrifuged at 2248× *g* for 10 min, the supernatant was discarded, and the protoplasts were diluted with 0.6 mol/L mannitol and stored at 4 °C for future use. A 0.01% Calcofluor White aqueous solution was added to the protoplast suspension, and an observation was conducted using an S-Ri2 Nikon fluorescence microscope (×100) with a DAPI filter [35].

Using a hemocytometer, the protoplast concentration was adjusted to 1 × 10^6^–1 × 10^7^ cells/mL under a light microscope. Then, 0.1 mL of protoplast suspension was spread onto a CYM regeneration plate (containing 2 g peptone, 4 g yeast extract, 20 g glucose, 0.5 g MgSO_4_, 0.48 g K_2_HPO_4_, 0.5 g H_2_PO_4_, 16 g agar, and 109 g mannitol). The protoplasts spread on the CYM plates were used as the control. The regeneration of protoplasts was observed after incubating at 28 °C for 7–10 days.

Protoplast regeneration rate (%) = (Number of colonies on CYM regeneration plate − Number of colonies on CYM plate)/Number of protoplasts × 100%

### 2.3. ARTP Mutagenesis of Protoplasts and Screening of Mutant Strains

ARTP mutagenesis was performed using an ARTP mutagenesis system (Wuxi Yuanqingtianmu Biotechnology Co., Ltd., Wuxi, China). The operating parameters were as follows: the radio frequency power input was 120 W, the distance between the plasma torch nozzle exit and the sample plate was 2 mm, and the helium gas flow rate was 10 SLM (standard liter per minute). For the mutation, 20 µL of the protoplast suspension was dipped into the stainless-steel plate and then exposed to the ARTP jet for 0, 20, 40, 60, 80, 120, 140, 160, and 200s, respectively. Three replicates were performed for each time treatment. The treated metal discs were placed in EP tubes containing 500 µL of 0.6 mol/L mannitol and shaken thoroughly. Then, 200 µL of the shaken solution was spread onto CYM regeneration medium and incubated at 28 °C for 7–10 days. The optimal mutagenesis treatment time was determined based on the survival rate curve calculated as follows: (control colony count − surviving colony count)/control colony count × 100%.

The control colony count was the total colony count of the untreated sample and the surviving colony count was the total colony count after the ARTP treatment.

The mutated protoplasts were diluted and spread onto CYM regeneration plates. After 10 d, small colonies appeared on the plates. The colonies with a faster growth rate and thicker hyphae were selected and inoculated onto CYM plates for further culture. After 15 days, the strains were transferred to PDA slant tubes for preservation and inoculated into the CYM seed liquid. After 4 days, the strains with different growth characteristics compared to the wild strain were inoculated into the fermentation medium and cultured for 10 days to measure the mycelial polysaccharide concentration and the mycelial dry weight. The mycelial morphology and fermentation broth color were recorded by photographing the seed liquid on the 7th day of cultivation. The mutant strains with significantly increased polysaccharide production were selected for genetic stability testing. The mutant strains were subcultured on plates every 15 d for a total of five generations, and the polysaccharide concentration and mycelial dry weight were measured for each generation (1st, 2nd, 3rd, 4th, and 5th generations) using the aforementioned methods to determine the genetic stability of the mutant strains.

### 2.4. Genomic Sequencing Analysis

Strain HS819 and mutant strain A27 were cultured to logarithmic growth phase. Mycelia were collected by filtration on filter paper and stored at −80 °C for DNA extraction. The mycelia (2 g) were lysed with 1% SDS solution. Impurities in the lysate were removed using phenol/chloroform/isoamyl alcohol extraction, and genomic DNA was precipitated with isopropanol. The recovered DNA was checked using agarose gel electrophoresis and quantified using the Qubit 2.0 fluorometer (Thermo Scientific, Waltham, MA, USA). The genomes of strain HS819 and mutant strain A27 were sequenced using Illumina NovoSeq technology (Beijing Novogene Science and Technology Co., Ltd., Beijing, China).

The genomes of strains HS819 and A27 were assembled using the SOAP v2.21 software, and the genes were predicted using Genemark-ES. Annotation was performed using the NCBI non-redundant protein sequence database (NR), KEGG database, and CAZy database. The genomes of strains HS819 and A27 were aligned using BWA v0.7.17 software to identify the mutation sites. Insertion–deletion sites were detected using GATK v4.0.5.1software and annotated using ANNOVAR(2015Mar22) software.

### 2.5. Statistical Analyses

All statistical analyses were performed using one-way ANOVA (post hoc Tukey test) in IBM SPSS Statistics 24.0, with a *p*-value < 0.05, which was considered significant.

## 3. Results

### 3.1. Assessment of Protoplast Regeneration Ability

**Figure 1:**
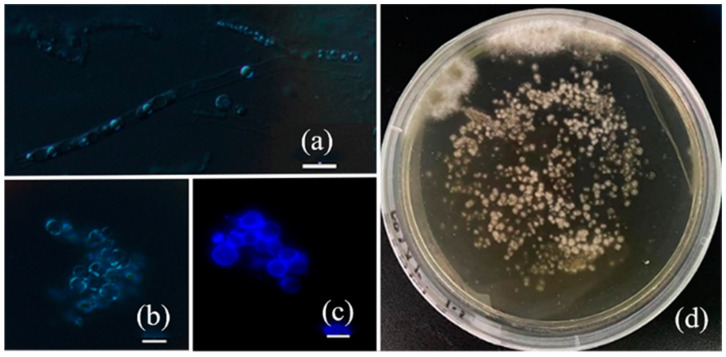
Morphology and regeneration of protoplasts. (**a**) Lateral release of the protoplast; (**b**) Protoplast of *I. obliquus*; (**c**) Protoplast of *I. obliquus* stained with Calcofluor White. Scale bar = 20 µm. (**d**) The morphology of the colonies of regenerated protoplast in CYM regeneration media.

Protoplasts are components of living cells that are obtained by removing the cell wall through mechanical or enzymatic methods. Under enzymatic hydrolysis conditions, factors such as mycelial growth time, enzyme concentration, and hydrolysis time can affect the protoplast quantity and quality. This experiment utilized 6-day-old mycelia of HS819 as the raw material, with 0.6 mol/L mannitol as the osmotic stabilizer, and a chitinase concentration of 30 mg/mL. The enzymatic hydrolysis was conducted at 30 °C for 1.5 h. Under these conditions, the mycelia primarily released protoplasts laterally (Figure 1a), with large voluminous protoplasts containing nuclei (Figure 1b). Mercaptoethanol can break the disulfide bonds in cell wall proteins and disrupt the protein protective layer outside of the cell wall [36]. Post-treatment, there was no significant change in protoplast yield after enzymatic hydrolysis of the mycelial slurry. Fluorescent staining of protoplasts was performed using Calcofluor White. Most protoplasts exhibited incomplete cell wall rupture, emitting blue fluorescence around the cell periphery (Figure 1c). The protoplasts were plated on CYM solid culture medium to observe the colony size and count, and the protoplast regeneration rate was calculated to be approximately 5.83% (Figure 1d). The absence of visible colony formation on control plates indicated that the prepared protoplasts were free from mycelial contamination.

### 3.2. Determination of Protoplast Mutagenesis Conditions

**Figure 2:**
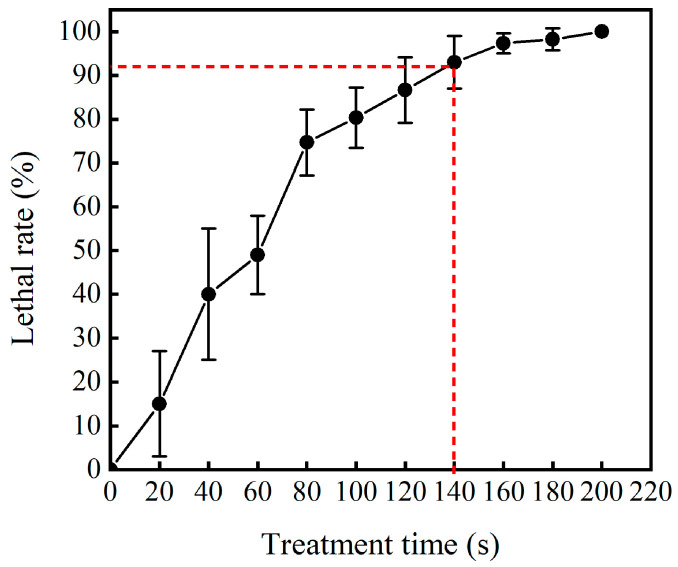
The relationship between mutagenesis time and lethality rate. The red dashed line indicates the treatment time selected for mutagenesis.

Under ARTP mutagenesis conditions with a power of 120 W, airflow of 10 SLM, temperature of 30 °C, and treatment time of 0–200 s, the lethality of the protoplasts increased with the prolongation of ARTP mutagenesis time, and no cells survived at 200 s (Figure 2). Studies have shown that, in ARTP mutagenesis experiments, a lethal dose with a lethality rate close to 90% is usually selected, rather than the highest lethal dose [37]. Typically, a survival rate of 10–20% is favorable for obtaining more positive mutants [38]. Therefore, the optimal duration of mutagenesis for protoplasts was 140 s, which resulted in a lethality rate of 93% for the HS819 protoplasts.

### 3.3. Screening of High-Yield Polysaccharide Mutants and Determination of Genetic Stability

**Figure 3:**
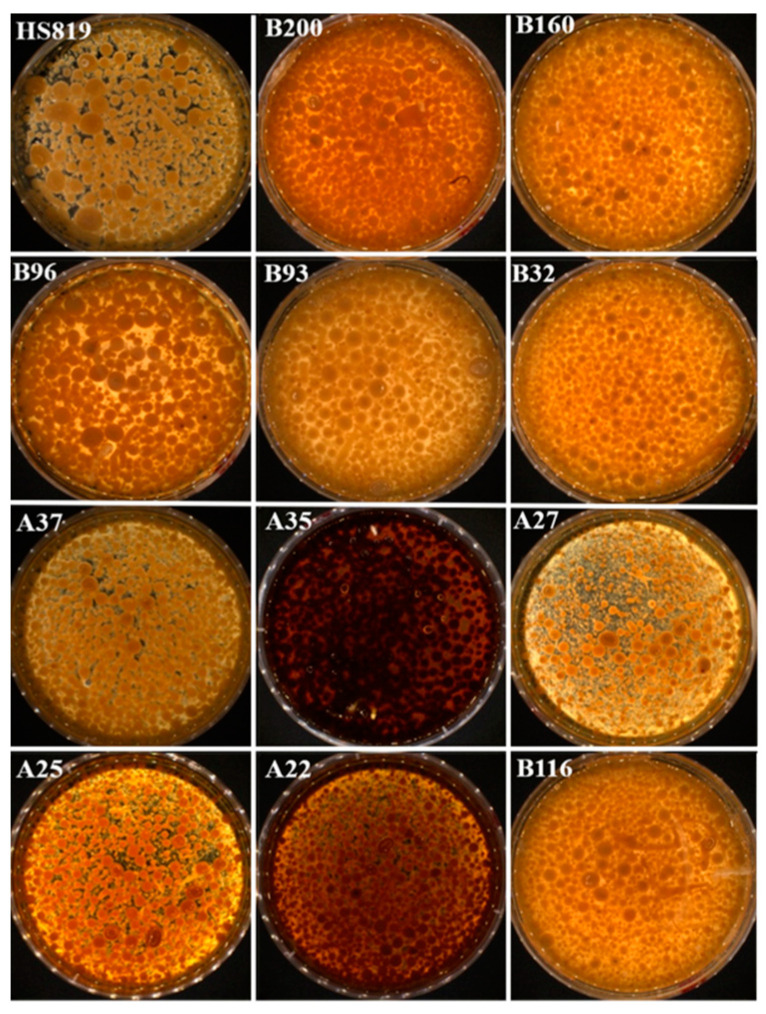
Morphology of mycelium pellets of some mutants.

**Figure 4:**
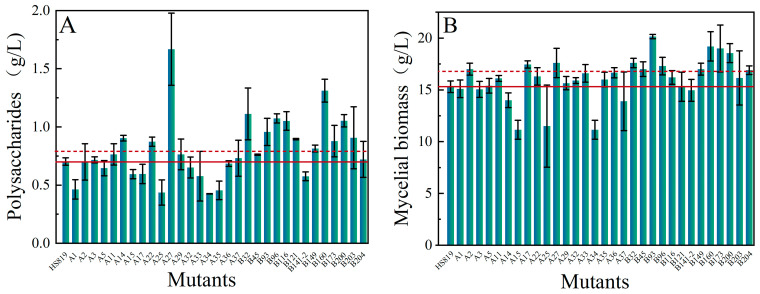
Mutant screening. (**A**) The yields of endo-polysaccharides of mutants. (**B**) The biomass of mutants. The red horizontal line represents the level of the original strain, whereas the red dashed line represents a 10% increase over the level of the original strain.

Among the 160 regenerated strains obtained from mutagenesis, 32 strains exhibiting better growth compared to the wild strain in the CYM seed liquid medium were preliminarily selected for liquid fermentation. All 32 selected strains showed certain phenotypic changes compared to the wild strain, mainly in growth rate, mycelial morphology, color of the fermentation broth, and biomass. The morphology of the mycelial pellets of some mutant strains is shown in Figure 3. Compared to the wild-type strain, 13 mutant strains showed polysaccharide levels (Figure 4A) and 11 mutant strains exhibited biomass levels (Figure 4B) exceeding those of the starting strain by 10% (the red horizontal line represents the level of the original strain, while the red dashed line represents a 10% increase over the level of the original strain). Among them, mutant strain A27 had a polysaccharide content as high as 1.67 g/L and a biomass of 17.6 g/L, representing an increase of 137.67% and 15%, respectively, compared to the wild strain. Moreover, mutant strains B116, B160, B200, B32, and B96 showed more than a 50% increase in polysaccharide production compared to the wild strain, with B200 and B160 exhibiting 21.24% and 25.49% higher biomass, respectively. Six dominant strains (A27, B116, B160, B200, B32, and B96) with high polysaccharide production were selected using shake flask fermentation. After five consecutive subcultures, the polysaccharide content and biomass of each mutant strain were measured to verify the genetic stability.

**Figure 5:**
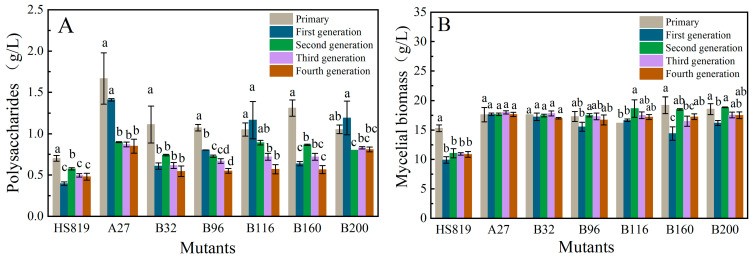
Genetic stability of the mutants. (**A**) Yields of polysaccharides of the mutants; (**B**) Biomass of the mutants. Note: Different lowercase letters indicate significant differences between generations for the same strain (*p* < 0.05).

Through shake flask fermentation, six dominant strains (A27, B116, B160, B200, B32, and B96) producing polysaccharides were screened. After five consecutive subcultures of these strains, the polysaccharide production and biomass of each generation of HS819 mutant strains were measured to verify their genetic stability. During the consecutive subculture process, except for strains B200 and A27, the polysaccharide production of the other mutant strains had already returned to the level of the original strains by the fifth generation, with the fifth generation of strain A27 showing higher production than B200 (Figure 5A). The biosynthesis of polysaccharides is not only associated with the metabolic characteristics of the strain itself, but also positively correlated with the biomass of the mycelium. For mutant strain A27, its biomass remained stable during the subculture process, and its stability was superior to that of mutant strain B200 (Figure 5B). Taking all these factors into consideration, mutant strain A27 was chosen for further investigation into polysaccharides.

### 3.4. Growth and Metabolic Curves in Liquid Cultures of Wild Strain HS819 and Mutant Strain A27

**Figure 6:**
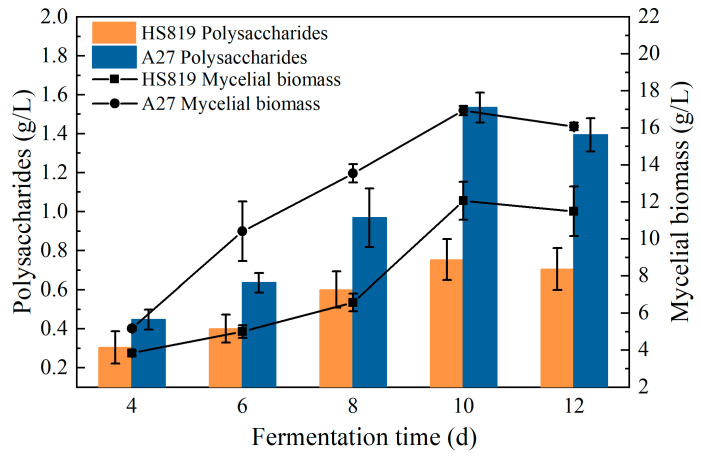
Polysaccharide and mycelial biomass curves of strain HS819 and mutant strain A27 during liquid fermentation.

The comparison of fermentation between wild strain HS819 and mutant strain A27 showed that, throughout the fermentation process, the trends in biomass and polysaccharide production of the two strains were similar, reaching their maximum values on the 10th day before entering a decline phase, with the mycelia undergoing autolysis (Figure 6). The polysaccharide concentration of mutant strain A27 began to increase rapidly from the 8th day, reaching 1.53 g/L at the end of fermentation, a 103.19% increase compared to the 0.753 g/L of the wild strain HS819. Additionally, the mutant strain exhibited a maximum biomass 20.5% higher than that of the wild strain, with biomass values of 16.93 g/L and 14.05 g/L, respectively. It is noteworthy that mutant strain A27 reached biomass levels equivalent to the wild strain on the 4th day of cultivation in the seed liquid, shortening the actual growth cycle by 2 days compared to the wild strain.

### 3.5. InDel Mining and Annotation of InDel Related to Polysaccharides Metabolism

Whole-genome resequencing was performed on wild strain HS819 and mutant strain A27. Compared to strain HS819, strain A27 had 3790 InDel mutations, including 2140 insertion mutations and 1650 deletion mutations (Table 1). In the CDS region, 204 InDel mutations were identified, including 127 frameshift mutations, 6 start codon mutations, 12 stop codon mutations, and 1 nonsense mutation, accounting for 1.891%, 0.089%, 0.179%, and 0.015% of the total CDS count, respectively. The remaining 0.864% of gene mutations had no significant impact on protein coding (Table 2). These InDel mutations involved 612 functional genes, and the mutation status and NR annotation results of the genes related to polysaccharides are shown in Table 3.

Most of the gene mutations related to carbohydrate metabolism in the NR annotation were also involved in the KEGG pathway associated with carbohydrate metabolism and CAZy clustering analysis, as follows: in the starch and sucrose metabolism pathway, the genes A2252, A3290, 6373, and A4713 of mutant strain A27 had mutations; and, in the glycolysis/gluconeogenesis pathway, the genes A6373, A1029, A3232, and A3715 had mutations. In addition, in the amino sugar nucleotide metabolism pathway, gene A6373 had mutations. The mutated A2408 and A4675 genes are involved in multiple pathways of N-glycan biosynthesis. In the interconversion pathway between mannose and glucuronic acid, gene A5969 had mutations. Furthermore, in the pentose phosphate pathway, gene A6373 had mutations. In the galactose metabolism pathway, the mutated gene was A4713, and, in other O-glycan biosynthesis pathways, gene A7152 had mutations (Table 4). The differential genes related to polysaccharide metabolism in the A27 mutant were also associated with glycoside hydrolases (GHs) activities, including A5969, A4713, A6734, A2408, A3290, A2252, and A3275. Gene A7545 was also associated with glycosyltransferases (GTs) activities (Table 5).

## 4. Discussion

The preparation and isolation of *I. obliquus* protoplasts are crucial for successful regeneration. In this study, 6-day-old HS819 mycelia were used as the raw material. The protoplast regeneration rate was approximately 5.83% using 0.6 mol/L mannitol as an osmotic stabilizer and 30 mg/mL of lytic enzyme at 30 °C for 1.5 h. Mercaptoethanol did not increase the enzymatic efficiency of the HS819 strain, which is consistent with the findings of Xu et al. [39], possibly due to the differences in cell wall composition, making it insensitive to mercaptoethanol. Although enzymatic hydrolysis is an effective method for protoplast preparation, steps such as filtration, washing, and low-speed centrifugation can lead to the loss and aggregation of suspended protoplasts, thereby affecting the final yield and regeneration rate of the protoplasts [23].

Xu et al. [40] reported a 37.7% increase in IPS1 and an 18.1% increase in IPS2 production 24 h after the addition of 0.1% (*v*/*v*) Tween 80, following the inoculation of *I. obliquus*. Wei et al. [41] achieved a 31% increase in mycelial polysaccharide production by implementing an integrated glucose titration and DO control strategy. Mutant strain JSU LIUK18, obtained through UV radiation mutagenesis, exhibited a 30.5% increase in mycelial polysaccharide compared to the wild-type strain [26]. These studies suggest that mycelial polysaccharide production in *I. obliquus* typically increases by approximately 30–50%, but the production efficiency still falls short of the market demand. This study, for the first time, utilized the atmospheric pressure and room temperature plasma (ARTP) mutagenesis breeding method with the original protoplasts of HS819 as the mutagenic material. Mutant strain A27 was selected from the mutagenized population with a cell death rate of 93%. Compared to parental strain HS819, mutant strain A27 showed a 137.67% increase in polysaccharides and a 15% increase in biomass. Additionally, the cultivation period was shortened from 14 days to 12 days, resulting in a significant improvement in production efficiency. ARTP technology has been widely used in bacterial, fungal, and microalgal mutagenesis to enhance productivity and improve characteristics [16,42,43]. Yang et al. [44] selected mutant 321 of Hericium erinaceus, with biomass increasing by 30.37% and intracellular polysaccharide content by 47.45%, compared to the original strain, using ARTP mutagenesis. Li et al. [45] screened a mutant of Sanghuangporus sanghuang via ARTP mutagenesis, wherein the average biomass of mutant strain A130 increased by 14.43%, compared with the original strain SH1, and the total polysaccharide contents of mutant strain A130 increased by 4.53%. Li et al. [46] used the protoplast of Ganoderma lucidum strain G0157 for ARTP treatment, obtaining mutant strain A-246, with the polysaccharide yield increasing by 268.57%, compared to the original strain. The results of this study are in accordance with previous studies, which have similarly reported the effective enhancement of polysaccharide production. These findings indicate that ARTP-mutagenesis-induced cellular genomic damage significantly alters the metabolic characteristics of the strain, particularly the polysaccharide production.

The increase in polysaccharide production of strain A27 may be attributed to the increase in biomass, the enhancement of polysaccharide-biosynthesis-related enzyme activities, and the upregulation of relevant gene expression levels. Genetic stability experiments have revealed that the mutation had some impact on the biomass of strain A27, but the increase in polysaccharide production was likely primarily caused by the other two factors. The mutated genes related to polysaccharide biosynthesis in the A27 strain included those encoding glucosylglucosephosphate mutase (A4175), phosphoglucoisomerase (A6373), glycosidase (A5969, A4173, A6734, A2408, A3290, A2252, and A3275), and glycosyltransferase (A7545). Among them, phosphoglucoisomerase (PGI) is involved in the pentose phosphate pathway, glycolysis/gluconeogenesis, and amino sugar and nucleotide sugar metabolism. Wang et al. [47] studied the biological function of PGI in *Lentinula edodes* and constructed a lepgi silencing strain using RNA interference technology, finding that the biomass of the lepgi silenced strain was significantly lower than that of the wild-type (WT) strain, and the levels of extracellular polysaccharides (EPS) and intracellular polysaccharides (IPS) increased by 1.5–3 times and 1.5 times, respectively. Phosphoglucomutase (PGM) is a key enzyme involved in polysaccharide synthesis, and some studies have shown that silencing the PGM gene is related to a decrease in extracellular polysaccharide production (about 20–40% of the WT strain) and an increase in intracellular polysaccharide production (about 1.7 times that of the WT strain) in *Ganoderma lucidum* [48]. Xu et al. [49] also showed that the overexpression of this gene can increase the maximum IPS content and EPS yield of *G.lucidum* by 40.5% and 44.3%, respectively. The GHs gene family regulates the high production of polysaccharides in *G.lucidum*. The expressed proteins (glycosidases) degrade the cell wall polysaccharides of hyphae, and some degraded polysaccharides are released into the culture medium, which is beneficial for the extraction of intracellular polysaccharides and can yield more extracellular polysaccharides [50]. Mannan polysaccharides are one of the components of fungal cell wall polysaccharides. The genome of strain A27 encodes genes for α-glucosidase (enzyme code ec3.2.1.20), α-galactosidase (enzyme code ec3.2.1.22), α-mannosidase (enzyme code ec3.2.1.113), β-glucosidase (enzyme code ec3.2.1.21), and β-galactosidase (enzyme code ec3.2.1.23), which are involved in the hydrolysis of the mannose skeleton, among which β-glucosidase and α-galactosidase are key enzymes in mannose skeleton hydrolysis [51]. The GTs gene family promotes the assembly of polysaccharides, and glycosyltransferases catalyze the continuous transfer of sugars from specific activated donors to specific receptor molecules, thereby forming region-specific and stereo-specific glycosidic bonds [52].

This study utilized ARTP and genome resequencing technology to analyze gene mutations related to high polysaccharide production in *I. obliquus*. The predicted polysaccharide-related mutated genes and specific mutated genes related to high polysaccharide production were analyzed. Further investigations should focus on changes at the transcriptional, expression, and metabolic levels to comprehensively elucidate that ARTP promotes high polysaccharide production in *I. obliquus*, providing valuable insights for obtaining high-yield, stable strains through genetic engineering and breeding.

## Figures and Tables

**Table 1:** Statistics of the total number of InDel in the samples.

Sample_Name	Insertion	Deletion	Total
A27	2140	1650	3790

**Table 2:** Statistics of the types of mutations caused by InDel in the CDS region.

Sample ID	Type	Frame-Shifted	Start Codon	Stop Codon	Premature Stop	Others Change	CDS_with_InDel	Total_CDS
A27	Number	127	6	12	1	58	204	6716
	Ratio(%)	1.891	0.089	0.179	0.015	0.864	3.038	

**Table 3:** Analysis of mutations in the polysaccharide-related differential gene of mutant strain A27 and NR annotation.

Gene_Id	InDel	Base	InDel Site	Subject_Id	NR Annotation
A1029	insertion	GAT	CDS	XP_007267446.1	succinate-semialdehyde dehydrogenase [Fomitiporia mediterranea MF3/22]
A2240	insertion	CGA	CDS	OCB87180.1	phosphoglycerate-mutase-like protein [Sanghuangporus baumii]
A2252	insertion	GAACTT	UTR	KIM22884.1	glycoside hydrolase family 1 protein [Serendipita vermifera MAFF 305830]
A2408	insertion	AAGCGTT	CDS	OCB89004.1	glycoside hydrolase [Sanghuangporus baumii]
A2798	insertion	A	CDS	XP_007271676.1	hexose transporter [Fomitiporia mediterranea MF3/22]
A3232	deletion	A	UTR	XP_007268233.1	NAD-aldehyde dehydrogenase [Fomitiporia mediterranea MF3/22]
A3275	deletion	T	UTR	XP_007263235.1	uncharacterized protein FOMMEDRAFT_131740 [Fomitiporia mediterranea MF3/22]
A3290	insertion	TGGCG	CDS	OCB89030.1	exo-beta-1,3-glucanase [Sanghuangporus baumii]
A3715	insertion	CGATTATCGC	UTR	OCB89533.1	NAD-dependent aldehyde dehydrogenase [Sanghuangporus baumii]
A4175	insertion	CTGGTG	CDS	XP_007263819.1	phosphoglucomutase 1 [Fomitiporia mediterranea MF3/22]
A4675	deletion	GCC	CDS	OCB91102.1	hypothetical protein A7U60_g1667 [Sanghuangporus baumii]
A4713	insertion	TGAGCTGGG	CDS	XP_007266231.1	uncharacterized protein FOMMEDRAFT_19850 [Fomitiporia mediterranea MF3/22]
A4748	deletion	CT	CDS	OCB90660.1	alcohol dehydrogenase [Sanghuangporus baumii]
A5969	deletion	A	CDS	XP_007262106.1	uncharacterized protein FOMMEDRAFT_164872 [Fomitiporia mediterranea MF3/22]
A6373	insertion	C	UTR	OCB86500.1	glucose-6-phosphate isomerase [Sanghuangporus baumii]
A7152	insertion	TCAAGGAG	CDS	OCB87804.1	O-mannosyltransferase [Sanghuangporus baumii]
A7545	insertion	CTCT	CDS	OCB87315.1	hypothetical protein A7U60_g5644 [Sanghuangporus baumii]

**Table 4:** KEGG enrichment analysis of mutated genes associated with polysaccharide production in the mutants.

Gene_Id	KEGG Gene_Id	KO_Id	Effect ^a^	Protein	Term
A6373	FOMMEDRAFT_26365	K01810	LOW	glucose-6-phosphate isomerase	Pentose phosphate pathway, Glycolysis/gluconeogenesis, Amino sugar and nucleotide sugar metabolism
A2252	Moror_16039	K01188	HIGH	beta-glucosidase	Starch and sucrose metabolism
A3290	FOMMEDRAFT_143134	K01210	HIGH	glucan 1,3-beta-glucosidase	Starch and sucrose metabolism
A4713	FOMMEDRAFT_19850	K01187	HIGH	alpha-glucosidase	Starch and sucrose metabolism
A1029	FOMMEDRAFT_109130	K14085	MODERATE	aldehyde dehydrogenase family 7 member A1	Glycolysis/gluconeogenesis
A3232	FOMMEDRAFT_89325	K00128	LOW	aldehyde dehydrogenase (NAD+)	Glycolysis/gluconeogenesis
A2408	FOMMEDRAFT_143158	K01230	HIGH	mannosyl-oligosaccharide alpha-1,2-mannosidase	Various types of N-glycan biosynthesis
A4175	FOMMEDRAFT_78059	-	HIGH		
A4675	HETIRDRAFT_146079	K03843	MODERATE	alpha-1,3/alpha-1,6-mannosyltransferase	Various types of N-glycan biosynthesis
A5969	FOMMEDRAFT_164872	K01213	LOW	galacturan 1,4-alpha-galacturonidase	Pentose and glucuronate interconversions
A4713	FOMMEDRAFT_19850	K01187	HIGH	alpha-glucosidase	Starch and sucrose metabolism
A7152	FOMMEDRAFT_16316	K00728	HIGH	dolichyl-phosphate-mannose-protein mannosyltransferase	Other types of O-glycan biosynthesis

^a^ Indicates the degree of impact of the mutation: HIGH > MODERATE > LOW.

**Table 5:** Analysis of CAZy data of mutated genes associated with polysaccharide production in mutant A27.

Gene_Id	GenBank_Accession	CAZy_Family	Effect ^a^	Family_Note
A5969	QRW19253.1	GH28	LOW	polygalacturonase (EC 3.2.1.15); exo-polygalacturonase (EC 3.2.1.67)
A4713	VWP01427.1	GH31	HIGH	alpha-glucosidase (EC 3.2.1.20); alpha-galactosidase (EC 3.2.1.22)
A6734	QRD92195.1	GH43_29	LOW	
A2408	QRW05345.1	GH47	HIGH	alpha-mannosidase (EC 3.2.1.113)
A3290	CAA63536.1	GH5_9	HIGH	
A2252	CCA69638.1	GH1	HIGH	beta-glucosidase (EC 3.2.1.21); beta-galactosidase (EC 3.2.1.23)
A3275	BCT97380.1	GH115	LOW	xylan alpha-1,2-glucuronidase (3.2.1.131); alpha-(4-O-methyl)-glucuronidase (3.2.1.-)
A7545	ADV25019.1	GT90	HIGH	UDP-Xyl: (mannosyl) glucuronoxylomannan/galactoxylomannan beta-1,2-xylosyltransferase (EC 2.4.2.-); UDP-Glc: protein O-beta-glucosyltransferase (EC 2.4.1.-);

^a^ Indicates the degree of impact of the mutation: HIGH > MODERATE > LOW.

## Data Availability

Dataset available upon request from the authors.
